# Human Herpesvirus 6 Encephalomyelitis

**DOI:** 10.3201/eid1009.040365

**Published:** 2004-09

**Authors:** Jose Luis Soto-Hernandez

**Affiliations:** * Instituto Nacional de Neurología Mexico, Mexico City, Mexico

**Keywords:** letter, herpesvirus, encephalomyelitis, CNS infections

**To the Editor:** Denes et al. (1) reports successful treatment of human herpesvirus 6 (HHV-6) encephalomyelitis. The patient was an immunocompetent young woman whose symptoms were fever, urinary retention, blurred vision, quadriparesis, bilateral papillitis, and optic neuritis. Magnetic resonance imaging (MRI) showed multiple lesions on the spinal cord white matter and the left thalamus, and the cerebral spinal fluid (CSF) showed inflammation. The patient was treated with acyclovir for 3 days, high-dose methylprednisolone for 5 days, cidofovir for 1 day, and ganciclovir for 15 days, starting on day 23 of hospitalization. By establishing a relationship between antiviral drug doses, serial determinations of HHV-6 DNA by polymerase chain reaction (PCR) in CSF, and neurologic improvement, Denes et al. concluded that antiherpesvirus drugs led to her recovery.

This case fits well in the spectrum of acute disseminated encephalomyelitis (ADEM), an inflammatory demyelinating disease of the central nervous systems of children and young adults, which occur in close temporal relationship with several infectious illnesses and immunizations (2–6). The disease has particular predilection to the optic nerves, spinal cord, brainstem, basal ganglia, and cerebral and cerebellar hemispheres. Maximal neurologic deficits are reached within several days, and resolution takes weeks or months. The condition is typically monophasic, but relapses have been reported (7). Histologic multifocal areas of inflammation and demyelinization are found. In the pathogenesis of ADEM, an initial injury caused by an infectious agent, followed by a secondary autoimmune response, has been postulated, and animal models have provided experimental support; both CD4 and CD8 T cells have been implicated in a secondary autoimmune response (6). Despite the lack of controlled studies, corticosteroids are widely used to treat ADEM and high-dose methylprednisolone is the drug of choice (3,4). The largest series of ADEM in adults included 40 patients with a mean follow-up period of 38 months. The patients were given a standardized treatment regimen of methylprednisolone, 500 mg daily intravenously for 5 days, with no additional therapy if they recovered completely. In patients with persistent neurologic deficits, the initial intravenous therapy was followed by a regimen of oral methylprednisolone, which was slowly tapered over 4 to 6 weeks. In patients with no response to this therapy, or whose condition had deteriorated during therapy, cyclophosphamide was given to seven patients, and immunoglobulin was given to one patient. Thirty-eight of 40 patients improved during the acute phase of the illness; in 7, symptoms completely resolved. One patient’s condition remained unchanged and one patient died; no antiviral drugs were given (5).

The neurotropism of HHV-6 and that the CNS may be a site of viral persistence or latency are well recognized (8,9). On autopsy, evidence of fulminant encephalitis with HHV-6 DNA demonstrated by PCR, immunohistochemical staining, or nucleic acid hybridization, confirms that HHV-6 causes acute CNS disease (8). Nevertheless, whether evidence of HHV-6 DNA in CSF demonstrated by PCR can be solely relied on is debatable. HHV-6 DNA was detected in the CSF of 41 (28.9%) of 142 children with a history of HHV-6 infection (9). HHV6-DNA was detected in the CSF of 47 (61%) of 77 children examined after primary HHV-6 infection. In the remaining 30 children (39%), HHV-6 DNA was detected in both peripheral blood mononuclear cells and CSF samples. HHV-6 variant A was detected more frequently in CSF than in specimens of other sites, which suggests that HHV-6A has greater neurotropism (10).

The role of HHV-6 in acute multifocal neurologic disease in immunocompetent adults requires additional observation, and its role in multiple sclerosis is in question. Much can be learned from careful study of patients (1).

I caution the casual reader who may conclude that using antiviral drugs against herpes viruses is recommended when acute mutlifocal neurologic disease clinically compatible with ADEM is indicated. High-dose IV methylprednisolone is the most utilized treatment, and the patient in the Denes et al. report was given it early in her hospital stay. The available evidence supports methylprednisolone as an essential drug in the management of ADEM.
