# Supplementary material for: Human Herpesvirus 6 Encephalomyelitis
Source: Emerg Infect Dis. 2004 Sep;10(9):1700–1. doi: 10.3201/eid1009.040365 (PMC3320276; doi:10.3201/eid1009.040365)
Supplement: Related texts

# Reply: Human Herpesvirus 6 Encephalomyelitis

**DOI:** 10.3201/eid1009.040522

**Authors:** Eric Denes, Sylvie Ranger-Rogez

**Affiliations:** * Centre Hospitalier Universitaire, Dupuytren, Limoges, France

**In Reply:** The disease that we reported (1) was encephalomyelitis induced by a human herpesvirus 6 (HHV-6) reactivation. Our aim was to emphasize that HHV-6 can cause such a disease, even when the patient is immunocompetent, and to urge physicians to search for it.

Implicating HHV-6 in the pathogenesis of neurologic manifestations in the reported case can be challenged, as suggested by Dr. Soto-Hernandez (2). Polymerase chain reaction (PCR) results must be interpreted cautiously, especially in cases that lack corroborating clinical evidence of infection. In our case, the diagnosis was made initially when HHV-6 was found in the patient's cerebral spinal fluid (CSF) by using PCR, by the absence of other cause, and by our experience; adult CSF is usually negative for HHV-6 by using PCR. Moreover, in our case, clinical symptoms and HHV-6 in the patient's CSF evolved in the same way. The neurologic tropism of HHV-6 is well known, and the main manifestation in adults is encephalitis, especially in an immunosuppressed context. Diagnosis is usually based on finding HHV-6 genetic material in the CSF (3,4), which has now replaced brain biopsy. Positive tissue results do not distinguish latent from productive infections when PCR-positive CSF indicates viral particle production in the central nervous system (CNS). In our case, the absence of brain tissue did not allow immunohistochemical staining or in situ hybridization. In the study by Caserta et al. (5), cited by Dr. Soto-Hernandez, HHV-6 PCR was positive in CSF and negative in peripheral blood mononuclear cells in 28.9% of children <3 years old with prior HHV-6 infection. These results provide evidence of HHV-6 persistence in the CNS; this phenomenon is now well recognized. Nevertheless, HHV-6 persistence after primary infection is quite different from reactivation in an immunocompetent adult. High-avidity anti-HHV-6 immunoglobulin G detected in the patient's serum when the symptoms started proved that our patient had been infected with HHV-6 previously.

We appreciate Dr. Soto-Hernandez's suggestion concerning acute demyelinating encephalomyelitis (ADEM) in our case. ADEM is an inflammatory demyelinating disease of the CNS, occurring mostly in children and rarely in young adults, soon after an infection or a vaccination. The disease is often associated with exanthema. A virus is often thought to be the cause, but viral symptoms are often not documented and rarely treated. ADEM may evolve into multiple sclerosis, and HHV-6 has been proposed as one of the causes of that condition (6). For example, multiple sclerosis developed in 14% of children and 35% of adults with ADEM in the study by Schwarz et al. (7).

Spontaneous improvement of ADEM is regularly reported; however, when treatment is needed, especially during the acute phase, steroid therapy is frequently used. In our case, corticosteroids did not affect the evolution of the patient's neurologic symptoms. Conversely, introducing the antiherpes drugs (cidofovir and ganciclovir) was followed by improved clinical signs and negative results for HHV-6 in the CSF by PCR. Corticosteroids likely influence inflammation associated with ADEM, but if ADEM is the result of a viral infection with persistent viral replication, steroids might be deleterious, allowing an increase in viral replication (8,9).

In conclusion, we think that viruses, particularly HHV-6, should be considered in ADEM, even in immunocompetent patients. In case of a positive result, antiviral treatment must be given, eventually in association with corticosteroids. We cannot recommend using corticosteroids alone because of the risk of spreading the infection.

## References

[1] Denes E, Magy L, Pradeau K, Alain S, Weinbreck P, Ranger-Rogez S. Successful treatment of human herpesvirus type 6 encephalomyelitis in immunocompetent patient [letter]. Emerg Infect Dis. 2004;10:729–31.1520087110.3201/eid1004.030587PMC3323089

[2] Soto-Hernandez JL. Human herpesvirus 6 encephalomyelitis [letter]. Emerg Infect Dis. 2004;10.1550341510.3201/eid1009.040365PMC3320276

[3] Yoshihara S, Kato R, Inoue T, Miyagawa H, Sashihara J, Kawakami M, Successful treatment of life-threatening human herpesvirus-6 encephalitis with donor lymphocyte infusion in a patient who had undergone human leukocyte antigen-haploidentical nonmyeloablative stem cell transplantation. Transplantation. 2004;77:835–8. 10.1097/01.TP.0000119603.59880.4715077022

[4] Wainwright MS, Martin PL, Morse RP, Lacaze M, Provenzale JM, Coleman RE, Human herpesvirus 6 limbic encephalitis after stem cell transplantation. Ann Neurol. 2001;50:612–9. 10.1002/ana.125111706967

[5] Caserta MT, Hall CB, Schnabel K, McIntyre K, Long C, Costanzo M, Neuroinvasion and persistence of human herpesvirus 6 in children. J Infect Dis. 1994;170:1586–9. 10.1093/infdis/170.6.15867996000

[6] Cermelli C, Berti R, Soldan SS, Mayne M, D'ambrosia JM, Ludwin SK, High frequency of human herpesvirus 6 DNA in multiple sclerosis plaques isolated by laser microdissection. J Infect Dis. 2003;187:1377–87. 10.1086/36816612717618

[7] Schwartz S, Mohr A, Knauth M, Wildemann B, Storch-Hagenlocher B. Acute disseminated encephalomyelitis. A follow up study of 40 adult patients. Neurology. 2001;56:1313–8.1137618010.1212/wnl.56.10.1313

[8] Hudnall SD, Rady PL, Tyring SK, Fish JC. Hydrocortisone activation of human herpesvirus 8 viral DNA replication and gene expression in vitro. Transplantation. 1999;67:648–52. 10.1097/00007890-199903150-0000210096517

[9] Black JB, Sanderlin KC, Goldsmith CS, Gary HE, Lopez C, Pellett PE. Growth properties of human herpesvirus-6 strain Z29. J Virol Methods. 1989;26:133–45. 10.1016/0166-0934(89)90143-22693479
